# Pelvic Lymph Node Dissection at the Time of Radical Prostatectomy: Extended? Of Course Not!

**DOI:** 10.1016/j.euros.2022.06.014

**Published:** 2022-08-19

**Authors:** Prasanna Sooriakumaran, Tushar A. Narain, Reena Davda

**Affiliations:** a Department of Urology, Digestive Disease & Surgery Institute, Cleveland Clinic London, London, UK; b Department of Uro-oncology, University College London Hospitals NHS Foundation Trust, London, UK; c Nuffield Department of Surgical Sciences, University of Oxford, Oxford, UK

The European Association of Urology (EAU) guidelines recommend performing extended pelvic lymph node dissection (e-PLND) in patients with high- and intermediate-risk prostate cancer patients when the estimated risk of positive lymph nodes exceeds 5% [1]. However, there is little evidence for any oncological benefit from e-PLND during radical prostatectomy (RP), while there is clear evidence of greater surgical complications including lymphocele, lymphorrhoea, lower limb lymphoedema, thromboembolism, and obturator nerve or ureteral injury [2]. Although the role of e-PLND during RP is well defined by EAU guidelines, many surgical experts do not adhere to these guidelines.

Here we discuss why the practice of e-PLND during RP is unnecessary, with no oncological benefit on the one hand and significant operative risk on the other. We critically appraise the available data.

Despite EAU guidelines giving strict indications for e-PLND, the practice of any PLND during RP and the extent of dissection are largely dependent on individual surgeon preference. An audit of the British Association of Urological Surgeons national database on patients with high-risk localised prostate cancer who underwent RP in 2014–2015 (*n* = 3196) showed that e-PLND was performed in only a minority (36.3%), limited templates were used almost as frequently (27.7%), and no nodal dissection performed in almost one-third of cases (32.7%) [3]. Even when e-PLND was performed, the median nodal counts were comparatively low (12 for open, 15 for laparoscopic, and 12 for robotic RP), suggesting a reluctance among surgeons to perform a true extended dissection. This surgical hesitancy was confirmed in a US randomised controlled trial (RCT) that showed little difference in nodal yields between limited PLND and e-PLND (12 vs 14) [4]. While lymph counts are a poor surrogate for the extent of PLND, the lack of significant difference in counts suggest that an extended template is not commonly performed by RP surgeons, even in the USA.

The apparent lack of adherence to guidelines in real-world practice may be because of a lack of clinical benefit and the known risks of e-PLND. A Korean study showed that e-PLND did not alter biochemical recurrence rates compared to limited PLND, although the median follow-up was only 36 mo and the analysis was subject to retrospective biases [5]. However, a recent, large, multi-institutional, multinational study in patients with intermediate- and high-risk prostate cancer failed to demonstrate improvements in biochemical recurrence–free, metastasis-free, and cancer-specific mortality–free survival rates among patients who underwent e-PLND, even at 10 yr after RP [6]. Many other studies have also failed to establish any oncological benefit of e-PLND in RP, although they are limited by the lack of true extended template dissections being performed. Hence, most studies that compare e-PLND to either no or limited PLND may be comparing slightly more PLND with slightly less or no PLND, making it difficult to assess any true oncological benefit.

What we do know from RCTs is that e-PLND is associated with significant increases in operative time, intraoperative complications, bleeding, and hospital stay. Lymphocele development occurred significantly more often after e-PLND compared to limited PLND (17% vs 8%), and clearly omitting PLND altogether would result in no increased operative time and, more importantly, no risk of lymphocele development [7]. In a well-conducted systematic review, five studies showed higher perioperative complication rates in the e-PLND group compared to the limited PLND group, while five other studies did not find any significant differences [8]. Similarly, the rate of lymphocele was significantly higher in the e-PLND group in four studies, while no differences were observed in four others. Lack of difference in complication rates between e-PLND and limited PLND is again most likely because of the lack of true extended dissection during e-PLND, with the resulting comparison of slightly more PLND with slightly less PLND. What is clear is that any PLND, regardless of dissection template, is associated with complications, with no evidence of any therapeutic benefit. The question is therefore not how extended should we go with our PLND but rather should we do it at all?

While some would argue that PLND during RP provides useful staging information, new imaging modalities such as prostate-specific membrane antigen (PSMA) positron emission tomography (PET) can identify small lymph nodes of a few millimetres that were previously not detectable via conventional imaging. While some lymphatic micrometastases are “missed” by PSMA PET (sensitivity 40%), patients can be treated with postoperative radiation (whole-pelvis radiotherapy) if or when these nodes become PSMA PET-positive without long-term consequences [9]. Ongoing studies are evaluating whether PSMA PET can therefore replace PLND for primary nodal staging given its detection of all but the smallest lymphatic metastases [10].

In conclusion, e-PLND in RP provides pathological nodal staging in RP patients, but whether this has any oncological significance has yet to be demonstrated. Patients at high risk of N1 disease are increasingly being offered novel imaging with PSMA PET, which is currently under investigation as a modality for primary nodal staging. While any postulated benefit therefore of e-PLND remains uncertain, what is apparent is its higher complication rates, including lymphocele, lymphorrhoea, lower limb lymphoedema, and thromboembolism. Therefore, PLND should not be used in the surgical management of prostate cancer as its risks outweigh any potential staging benefit.

  ***Conflicts of interest***: The authors have nothing to disclose.

## References

[1] Mottet N., van den Bergh R.C.N., Briers E. (2021). EAU-EANM-ESTRO-ESUR-SIOG guidelines on prostate cancer—2020 update. Part 1: screening, diagnosis, and local treatment with curative intent. Eur Urol.

[2] Tewari A., Sooriakumaran P., Bloch D.A., Seshadri-Kreaden U., Hebert A.E., Wiklund P. (2012). Positive surgical margin and perioperative complication rates of primary surgical treatments for prostate cancer: a systematic review and meta-analysis comparing retropubic, laparoscopic, and robotic prostatectomy. Eur Urol.

[3] Aning J.J., Reilly G.S., Fowler S., Challacombe B., McGrath J.S., Sooriakumaran P. (2019). Perioperative and oncological outcomes of radical prostatectomy for high-risk prostate cancer in the UK: an analysis of surgeon-reported data. BJU Int.

[4] Touijer K.A., Sjoberg D.D., Benfante N. (2021). Limited versus extended pelvic lymph node dissection for prostate cancer: a randomized clinical trial. Eur Urol Oncol.

[5] Kim K.H., Lim S.K., Kim H.Y. (2013). Extended vs standard lymph node dissection in robot-assisted radical prostatectomy for intermediate-or high-risk prostate cancer: a propensity-score-matching analysis. BJU Int.

[6] Preisser F., van den Bergh R.C., Gandaglia G. (2020). Effect of extended pelvic lymph node dissection on oncologic outcomes in patients with D’Amico intermediate and high risk prostate cancer treated with radical prostatectomy: a multi-institutional study. J Urol.

[7] Schwerfeld-Bohr J., Kaemper M., Krege S., Heidenreich A. (2014). 747 Prospective randomized multicenter study comparing limited vs extended pelvic lymphadenectomy in intermediate and high risk prostate cancer–first descriptive results (SEAL, AUO AP 55/09). Eur Urol Suppl.

[8] Fossati N., Willemse P.P., Van den Broeck T. (2017). The benefits and harms of different extents of lymph node dissection during radical prostatectomy for prostate cancer: a systematic review. Eur Urol.

[9] Hope T.A., Elber M., Armstrong W.R. (2021). Diagnostic accuracy of 68-gallium-PSMA-11 PET for pelvic nodal metastasis detection prior to radical prostatectomy and pelvic lymph node dissection: a multicentre prospective phase 3 imaging trial. JAMA Oncol.

[10] Klingenberg S., Jochumsen M.R., Ulhøi B.P. (2021). ^68^Ga-PSMA PET/CT for primary lymph node and distant metastasis NM staging of high-risk prostate cancer. J Nucl Med.

